# Insights into the Mechanochemical Synthesis of MOF-74

**DOI:** 10.1021/acs.cgd.1c00213

**Published:** 2021-04-27

**Authors:** Jethro Beamish-Cook, Kenneth Shankland, Claire A. Murray, Paz Vaqueiro

**Affiliations:** † School of Chemistry, Food and Pharmacy, University of Reading, Whiteknights, Reading RG6 6DX, United Kingdom; ‡ Diamond Light Source, Harwell Science and Innovation Campus, Didcot OX11 0DE, United Kingdom

## Abstract

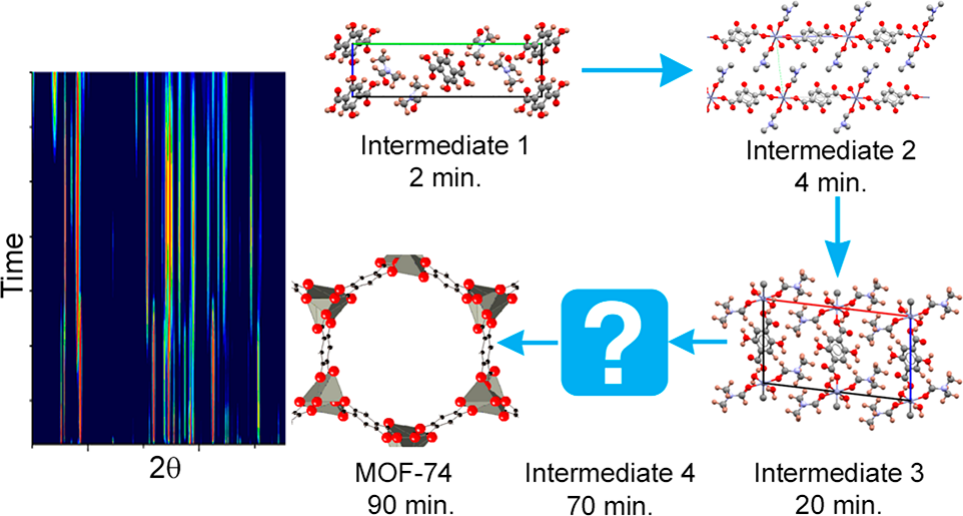

Mechanochemical synthesis has recently emerged as a scalable “green” approach for the preparation of MOFs, but current understanding of the underlying reaction mechanisms is limited. In this work, an investigation of the reaction pathway of the mechanochemical synthesis of MOF-74 from ZnO and 2,5-dihydroxyterephthalic acid (H_4_HDTA), using DMF as a liquid additive, is presented. The complex reaction pathway involves the formation of four short-lived intermediate phases, prior to the crystallization of MOF-74. The crystal structures of three of these intermediates have been determined using a combination of single-crystal and powder X-ray diffraction methods and are described here. The initial stages of the reaction are very fast, with a DMF solvate of H_4_HDTA forming after only 2 min of milling. This is followed by crystallization, after only 4 min of milling, of a triclinic one-dimensional coordination polymer, Zn(H_2_DHTA)(DMF)_2_(H_2_O)_2_, which converts into a monoclinic polymorph on additional milling. Highly crystalline MOF-74 appears after prolonged milling, for at least 70 min.

## Abstract 2

The mechanochemical synthesis of MOF-74 from ZnO and 2,5-dihydroxyterephthalic acid, using DMF as a liquid additive, has been investigated. The reaction pathway of involves the formation of four short-lived intermediate phases prior to the crystallization of the final product, MOF-74.

## Introduction

Metal–organic frameworks (MOFs) are a fascinating class of porous materials with a host of potential applications, ranging from drug delivery^1−4^ to catalysis^5−7^ and gas separation and storage.^8−10^ In academic research, MOF synthesis is dominated by solvothermal methods, which offer low space–time yields and often require expensive organic solvents,^11^ making them unsuitable for industrial production. The development of synthetic strategies that enable industrial manufacturing of MOFs at the required scale and cost is essential for the exploitation of these materials in new technologies.^12^ Mechanochemistry, a synthetic approach in which chemical reactions occur by grinding or milling in the absence of or with little solvent,^13,14^ has emerged as a scalable “green” alternative to solvothermal synthesis. Mechanochemical syntheses of several archetypal MOFs, including ZIFs,^15^ MOF-5,^16^ MIL-100,^17^ MOF-74,^18,19^ HKUST-1,^20^ and UiO-66^21^ have already been described, demonstrating the broad applicability of this synthetic approach. High-quality MOFs, with specific surface areas comparable to those prepared by conventional approaches, can be produced in quantitative yields by ball milling.^20,21^

An understanding of the underlying mechanisms by which mechanochemical reactions proceed is still limited. Key aspects of reactivity, such as the reaction kinetics or the effect of temperature upon the reaction, have been largely unexplored. Powder X-ray diffraction can be used to monitor the structural transformations that take place during mechanochemical syntheses, which can involve intermediates different from those observed in conventional syntheses. For instance, during the mechanochemical synthesis of ZIF-8 from ZnO and 2-methylimidazole, amorphization is observed, followed on further milling by crystallization of a new metastable phase with a katsenite (“kat”) topology.^22^ The reaction rates appear to be strongly temperature dependent,^23^ and it has been shown in a model system (a cadmium coordination polymer) that modest changes in temperature can result in changes in the reaction mechanism.^24^ Small amounts of liquid additives can also influence the pathway of the reaction, as exemplified by the synthesis of HKUST-1, for which two previously undetected intermediate phases containing a mononuclear copper core have been identified, depending on the milling conditions and the additives used.^25^ Although equipment suitable for *in situ* powder diffraction experiments at synchrotron beamlines has been developed,^26,27^ the identification of reaction intermediates based on powder diffraction patterns of complex reaction mixtures can be challenging. The Cambridge Structural Database or the Inorganic Crystal Structure Database can be used for the identification of previously reported phases,^26^ while for previously unreported phases, structure-solution methods using powder diffraction data can be exploited.

Given that MOF-74 is attracting much interest as a highly efficient material for the separation of CO_2_ and harmful gases (e.g., SO_2_, NH_3_)^28,29^ and that according to modeling studies it may also outperform traditional nanoporous drug storage materials,^30,31^ understanding the mechanism by which MOF-74 forms by ball milling is essential. It has been noted that intermediates are formed during the mechanochemical synthesis of MOF-74 using water and DMF as additives, but their structural characterization remains incomplete.^18^ Here, we present a detailed structural study of the intermediates formed during the synthesis of MOF-74 using DMF as a liquid additive. We demonstrate that the mechanosynthesis of MOF-74 is a complex process, involving the formation of four intermediate phases, three of which are fully characterized here, and where the nature of the liquid additive plays a major role.

## Experimental Section

All reagents, with the exception of 2,5-dihydroxyterephthalic acid (H_4_HDTA) that was purchased from Alfa Aesar, were obtained from Sigma-Aldrich and used without further purification.

### Monitoring of the Mechanochemical Synthesis of MOF-74

Zinc oxide (0.181 g, 2.22 mmol) and H_4_DHTA (0.218 g, 1.15 mmol) were placed in a 25 mL stainless steel grinding jar, together with a single 7 g stainless steel grinding ball and 250 μL of DMF. The jar was sealed and milled at a rate of 30 Hz using a Retsch MM400 shaker-type mixer mill, and small portions of the powder (5–10 mg) were taken at different times (2, 4, 6, 8, 10, 12, 14, 20, 30, 40, 50, 60, 70, 80, and 90 min) throughout the milling process. Initial screenings of the reaction were carried out using a Bruker D8 Advance instrument operating in transmission capillary mode (monochromatic Cu Kα_1_ radiation). Once the reaction timings had been established, powder X-ray diffraction data were collected at the Diamond Light Source I11 high-resolution powder diffraction beamline, using an energy of 8.976 keV. The wavelength and detector zero point were calibrated using a Si standard and found to be 1.381246(10) Å and −0.00971(3)° 2θ, respectively. Data were collected using five MAC (multi analyzer crystal) detectors. Immediately after each sample was extracted from the mill, the powder was loaded into a 0.7 mm diameter borosilicate glass capillary, which was mounted directly on the goniometer of the beamline. Samples were rotated at 300 rpm during data collection. To reduce the possibility of radiation damage, samples were translated by 3 mm every 5 min, with total collection times of 30 min per sample. Data were rebinned to an equivalent step size of 0.003° 2θ. The optimal reaction conditions for the preparation of MOF-74 can be found in the Supporting Information.

### Structural Determination

The structural characterization of Intermediate **1** was carried out using the I11 diffraction data collected as described above. However, most of the powder patterns collected when the mechanochemical synthesis was monitored correspond to mixtures of phases. These powder patterns enabled the identification of intermediates **2**–**4**, but not their complete structural characterization. Given the presence of unreacted ZnO in these patterns, additional mechanochemical reactions, with different ZnO:H_4_DHTA molar ratios, were carried out. Attempts to grow single crystals were also carried out. The optimized syntheses of each reaction intermediate to enable structural determination are described below, together with the approach adopted in each case for structural characterization. Selected crystallographic information for all identified phases is presented in Table 1. The crystal structures of intermediates **1**–**3** have been deposited at the Cambridge Crystallographic Data Centre, with deposition numbers CCDC 2063893–2063895.

**Table 1:** Selected Crystallographic Information for the Intermediates of the Mechanochemical Synthesis of MOF **74**

	intermediate **1**	intermediate **2**	intermediate **3**	intermediate **4**
solution method	powder diffraction	single crystal	powder diffraction	powder diffraction
empirical formula	C_14_H_20_N_2_O_8_	C_14_H_22_N_2_O_10_Zn	C_14_H_22_N_2_O_10_Zn	not known
formula wt	344.32	443.73	443.73	not known
temp (K)	293	250(3)	293	293
cryst syst	monoclinic	triclinic	monoclinic	triclinic
space group	*P*2_1_/*n*	*P*1̅	*P*2_1_/*c*	*P*1̅
*a* (Å)	5.92029(5)	5.415(5)	10.01162(6)	17.201(5)
*b* (Å)	20.8177(2)	8.709(5)	5.41376(3)	14.317(6)
*c* (Å)	6.87864(8)	10.118(5)	17.55111(11)	7.423(7)
α (deg)	90	82.786(5)	90	114.87(3)
β (deg)	98.0970(7)	89.035(5)	96.8507(7)	88.88(9)
γ (deg)	90	78.215(5)	90	110.31(5)
*V* (Å^3^)	839.319(15)	463.4(6)	944.487(10)	1538.06(4)
*Z*	2	1	2	not known
radiation (Å)	1.381246	1.5406	1.381246	1.5406
no. of indep rflns	724	1689	381	
no. of data/restraints/params	51 params	1689/0/148	53 params	
goodness of fit on *F*^2^	n/a	1.047	n/a	
final *R* indexes (*I* ≥ 2σ(*I*))		R1 = 0.0344, wR2 = 0.0803		
*R* factors (Pawley, Rietveld) (%)	5.36, 7.17		8.01, 9.93	

### Equipment and Methods for Structural Characterization

Single-crystal diffraction data were collected at 150 K on an Oxford Diffraction Gemini instrument equipped with a liquid-N_2_-based Cryojet cooling device, using a Kα monochromated copper source (λ = 1.5406 Å). Data were collected and reduced using CrysAlisPro.^32^ Structures were solved using SHELXT^33^ and refined using SHELXL,^34^ operating within the Olex2^35^ software package.

Powder X-ray diffraction data were indexed using the DASH^36^ and TOPAS 4.2^37^ software suites. Pawley and Rietveld refinements were performed using TOPAS 4.2. Structure solution from powder diffraction data was achieved using either the DASH or EXPO^38^ package. In cases where multiple crystalline phases were present in the data, individual crystalline phases were fitted with a mixture of Pawley (for indexed phases with unknown atomic coordinates) and Rietveld (for known phases) refinements, allowing the contribution of the unknown phase to be extracted from the observed data. For all structures that were solved from powder diffraction data, energy minimization of the determined structure was carried out through DFT calculations, implemented using the Quantum Espresso program PWscf v6.3.^39^ Following DFT optimization, rigid-body Rietveld refinements were carried out in order to generate the final crystal structure.

### Structural Determination of Intermediate **1**: (H_4_DHTA)(DMF)_2_

The powder pattern collected after 2 min of milling corresponded to a mixture of ZnO and intermediate **1**. The crystal structure of this intermediate was solved by first using EXPO to obtain a partial structure that revealed the unit cell contents and then DASH for the final structure solution, using half a molecule of H_4_DHTA anchored around a center of symmetry and one molecule of DMF (a total of nine degrees of freedom) as the input to the global optimization structure-solving process.^40^

### Synthesis and Structural Determination of Intermediate **2**: Zn(H_2_DHTA)(DMF)_2_(H_2_O)_2_

ZnO (180 mg, 2.2 mmol) and H_4_DHTA (220 mg, 1.1 mmol) were placed in a 25 mL milling jar along with 450 μL of DMF and a single 7 g milling ball. The jar was sealed, the contents were milled for 15 min, and then the jar was left sealed for 4 days. At the end of the 4 days, the jar was opened and small single crystals were found. Single-crystal diffraction data were collected on a small platy crystal with dimensions 0.1 × 0.1 × 0.05 mm.

### Synthesis and Characterization of Intermediate **3**: Zn(H_2_DHTA)(DMF)_2_(H_2_O)_2_

Zinc acetate dihydrate (137.9 mg, 0.75 mmol) and H_4_DHTA (144.584 mg, 0.73 mmol) were placed in a 25 mL stainless steel grinding jar along with two 4 g stainless steel grinding balls and 150 μL of DMF. The jar was sealed, and the contents were milled for 60 min at a rate of 30 Hz. An analysis of synchrotron powder diffraction data collected on the product indicated that this is a mixture of Zn(H_2_DHTA)(H_2_O)_2_ (CCDC refcode ODIPOH)^18^ and intermediate **3**. Following indexing of the non-ODIPOH peaks, the diffraction intensities for intermediate **3** were extracted using TOPAS and its crystal structure was solved using EXPO.

### Synthesis of Intermediate **4**

Zinc oxide (178.8 mg, 2.11 mmol) and H_4_DHTA (220.1 mg, 1.05 mmol) were placed in a 25 mL stainless steel milling jar along with a single 7 g stainless steel milling ball and 250 μL of DMF. The jar was sealed and heated to 60 °C for 60 min. The heated sample was milled for 5 min at 30 Hz to produce a mixture of intermediate **4** and zinc oxide. The structural characterization of intermediate **4** was not possible.

## Results and Discussion

**Figure 1:**
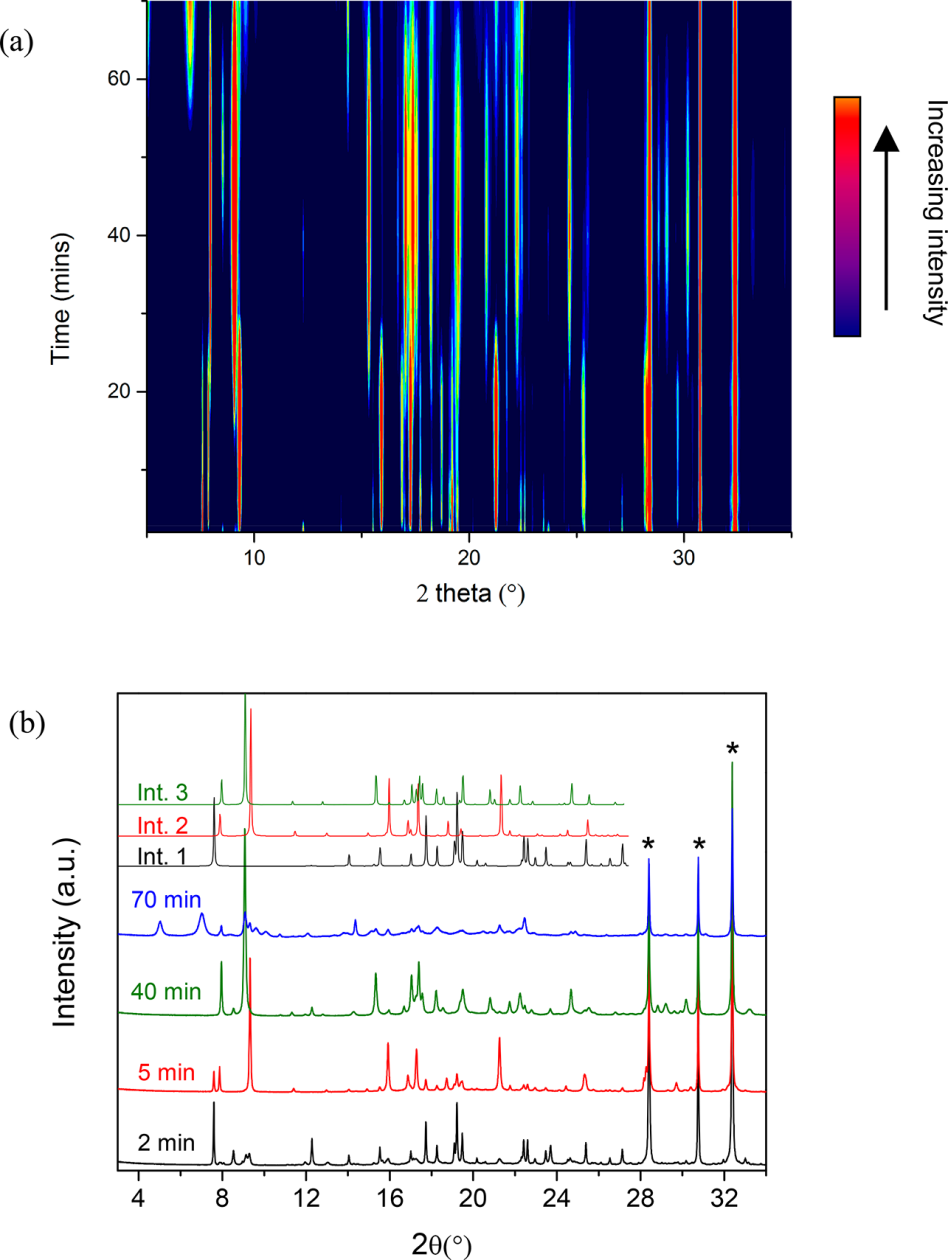
(a) Waterfall plot of the I11 powder diffraction data (λ = 1.381246 Å) of the milling of H_4_DHTA and ZnO (1:2 ratio) in the presence of a small amount of DMF. Each individual powder pattern was collected for 30 min. (b) Selected powder diffraction patterns, showing the presence of each intermediate. ZnO peaks have been labeled with a star. Simulated powder patterns for Intermediates **1**–**3** are shown at the top.

High-resolution powder X-ray diffraction data collected as a function of milling time enabled the identification of four short-lived reaction intermediates, all of which degrade in a matter of hours/days after preparation. The formation of each intermediate is evident in Figure 1, where large changes in peak intensities, with numerous diffraction peaks appearing and disappearing, can be observed as a function of time. The initial steps of the reaction occur very quickly, with the formation of intermediate **1**, which we subsequently established is a DMF solvate of H_4_DHTA, occurring after only 2 min of grinding. Intermediate **2**, which contains Zn and H_2_DHTA in a 1:1 ratio, starts to form after 4 min of grinding, while intermediate **3**, which is a polymorph of intermediate **2**, appears after milling for 20 min. Another phase, intermediate **4**, is first observed after 50 min, while MOF-74, which contains Zn and the organic linker in a 2:1 ratio, finally appeared after 70 min of milling. As each powder pattern corresponds to a mixture of phases and the composition of these previously unreported intermediates was not known, the structural characterization of the intermediates was challenging and required a combination of direct and simulated-annealing-based powder diffraction methods together with single-crystal diffraction.

### Crystal Structure of Intermediate **1**

**Figure 2:**
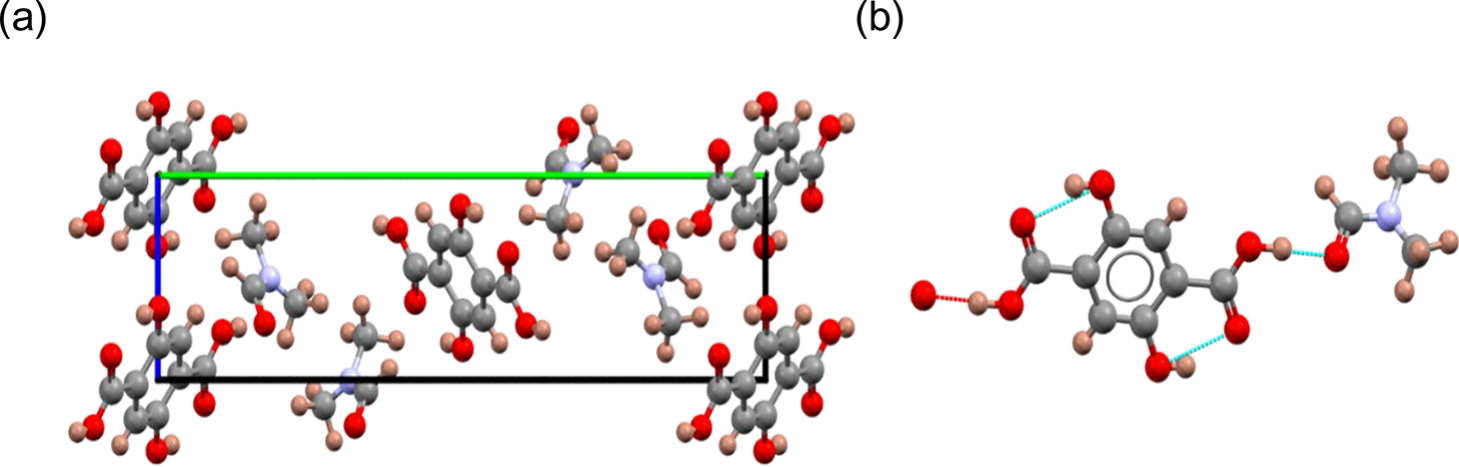
Crystal structure of intermediate **1**: (a) view of the unit cell along the *c* axis; (b) inter- and intramolecular H-bonds, shown as blue dotted lines, between neighboring H_4_DHTA and DMF molecules. Color key: carbon, gray spheres; oxygen, red spheres; nitrogen, blue spheres; hydrogen, pink spheres.

The asymmetric unit of intermediate **1**, which crystallizes in the monoclinic space group *P*2_1_/*n* (Table 1), contains half a H_4_DHTA molecule and one DMF molecule (Figure 2). The H_4_DHTA molecules pack in layers parallel to the (010) planes, and the DMF molecules are located between the H_4_DHTA layers. Throughout the crystal structure, the H_4_DHTA molecules exhibit both inter- and intramolecular hydrogen bonding. Short distances of 1.679(4) Å between the hydroxyl and carboxyl groups of the linker are consistent with intramolecular hydrogen bonding, while intermolecular hydrogen bonding distances of 1.502(6) Å are observed between carboxyl groups in the H_4_DHTA molecules and DMF. The final rigid-body multiphase Rietveld refinement (*R*_wp_ = 7.17%) is included in Figure S2 in the Supporting Information.

### Crystal Structure of Intermediate **2**

**Figure 3:**
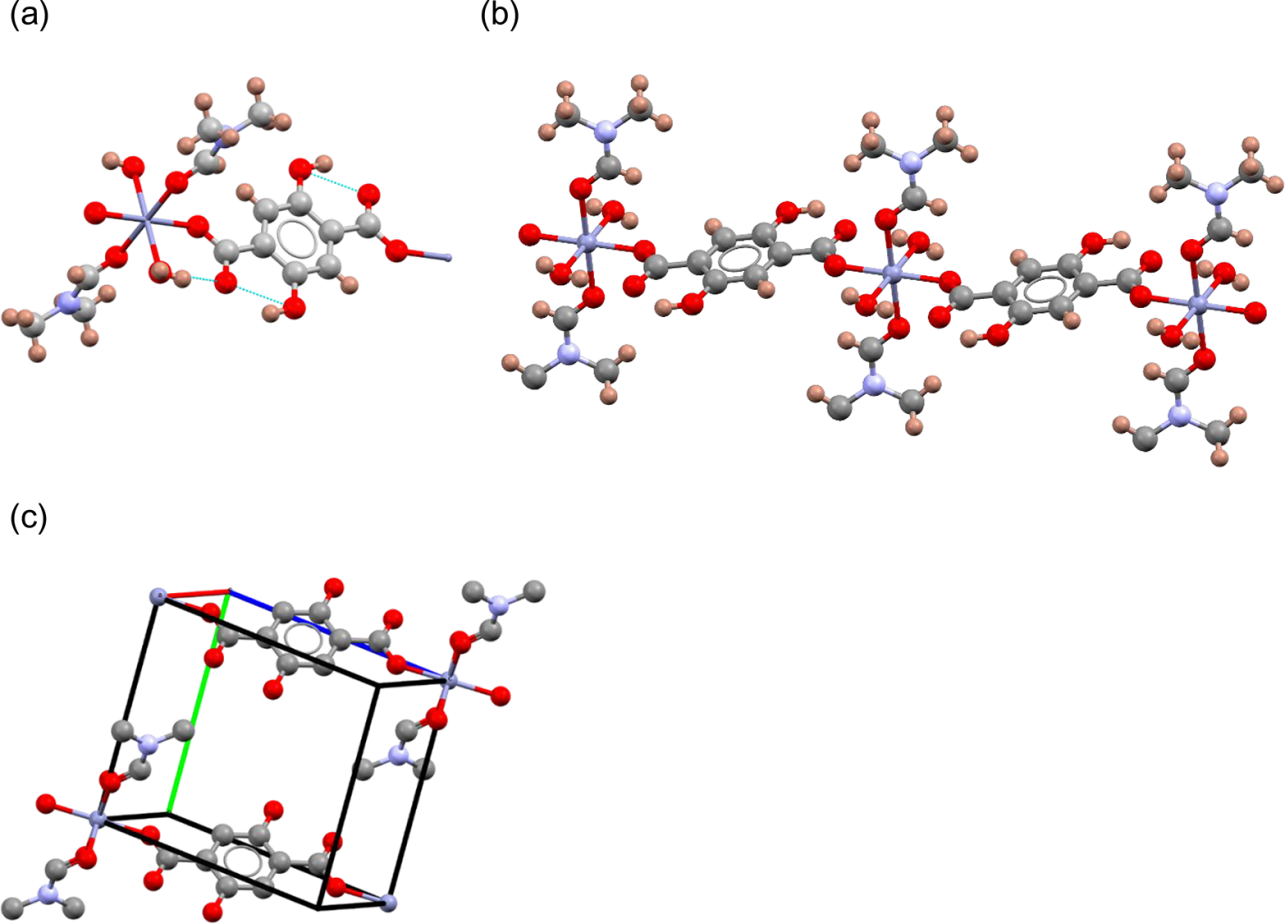
Crystal structure of intermediate **2**: (a) coordination around the octahedral zinc center, illustrating the hydrogen bonding; (b) view of a one-dimensional chain; (c) view of the unit cell, with hydrogen atoms omitted for clarity, showing the packing of the chains. Color key: zinc, blue spheres; carbon, gray spheres; oxygen, red spheres; nitrogen, pale blue spheres; hydrogen, pink spheres.

Intermediate **2**, which crystallizes in the triclinic space group *P*1̅ (Table 1), contains zinc and the linker in a 1:1 molar ratio. The asymmetric unit (Figure 3a) contains one zinc atom, one DMF molecule, one water molecule, and half an H_2_DHTA linker. Zinc is octahedrally coordinated to two water molecules, two DMF molecules, and two H_2_DHTA linkers, all in a *trans* arrangement. The Zn–O distances range between 2.0627(18) and 2.141(2) Å, with the shortest distances being to the linker and the longest to the water molecules. Each carboxylate group in the linker exhibits monodentate coordination to a zinc metal center. As each H_2_DHTA linker is coordinated to two Zn centers, the structure contains one-dimensional chains (Figure 3b). A relatively short O···H distance of 1.84(4) Å is indicative of intramolecular hydrogen bonding between the hydroxyl group and the carboxylate in the linker, with additional hydrogen bonding occurring between water molecules and the carboxylate groups. There is no evidence of hydrogen bonding between the chains. As illustrated in Figure 3c, all chains exhibit the same alignment, with no offset between neighboring chains, and there is a zinc–zinc distance of 8.709(5) Å between chains. A solution-based method for the preparation of this compound has been reported very recently.^41^

### Crystal Structure of Intermediate **3**

**Figure 4:**
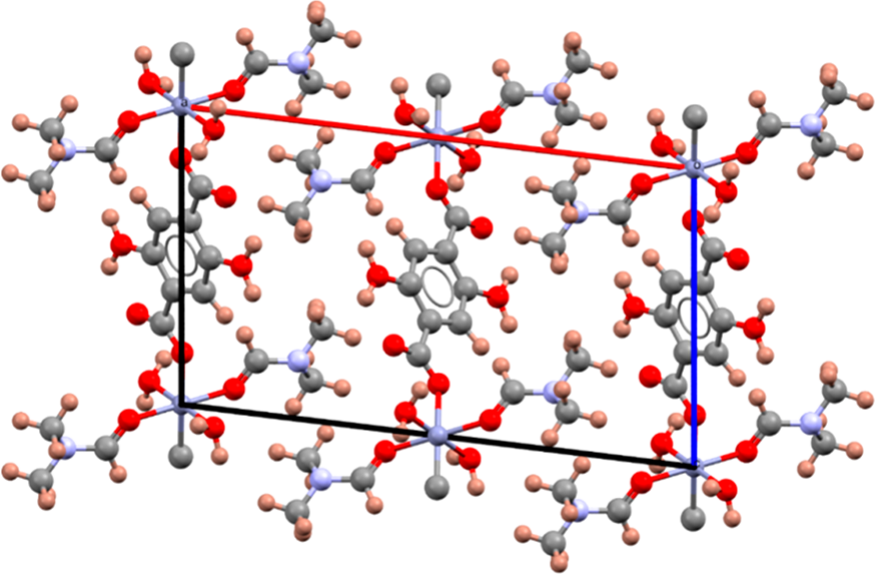
View of the crystal structure of intermediate **3** along the *b*-axis. The color key is given in the caption to Figure 3.

Intermediate **3**, which crystallizes in the monoclinic space group *P*2_1_/*c* (Table 1), is a polymorphic form of the same material that comprises intermediate **2**. Intermediate **3** forms upon further milling of intermediate **2** or by milling zinc acetate dihydrate with H_4_DHTA in a 1:1 ratio for 90 min. The local coordination environment around the zinc atom (Figure 4) is identical with that found for intermediate **2**, and the structure also contains one-dimensional chains, with all of the ligands in a *trans* arrangement. However, while in intermediate **2** all chains exhibit the same orientation, in the crystal structure of this intermediate the chains are packed in layers parallel to the (001) planes, with an ABAB··· stacking sequence along the *c* axis. All chains in a given layer are arranged in the same orientation, and layers where the chains are aligned along the [110] direction alternate with layers where the chains are oriented along [−110]. The final rigid-body multiphase Rietveld refinement for this intermediate has been included in Figure S3 in the Supporting Information. An isostructural cobalt compound, Co(H_2_DHTA)(DMF)_2_(H_2_O)_2_,^42^ has been subsequently identified using the Cambridge Structural database (CSD refcode SEWXIE).

### Intermediate **4**

**Figure 5:**
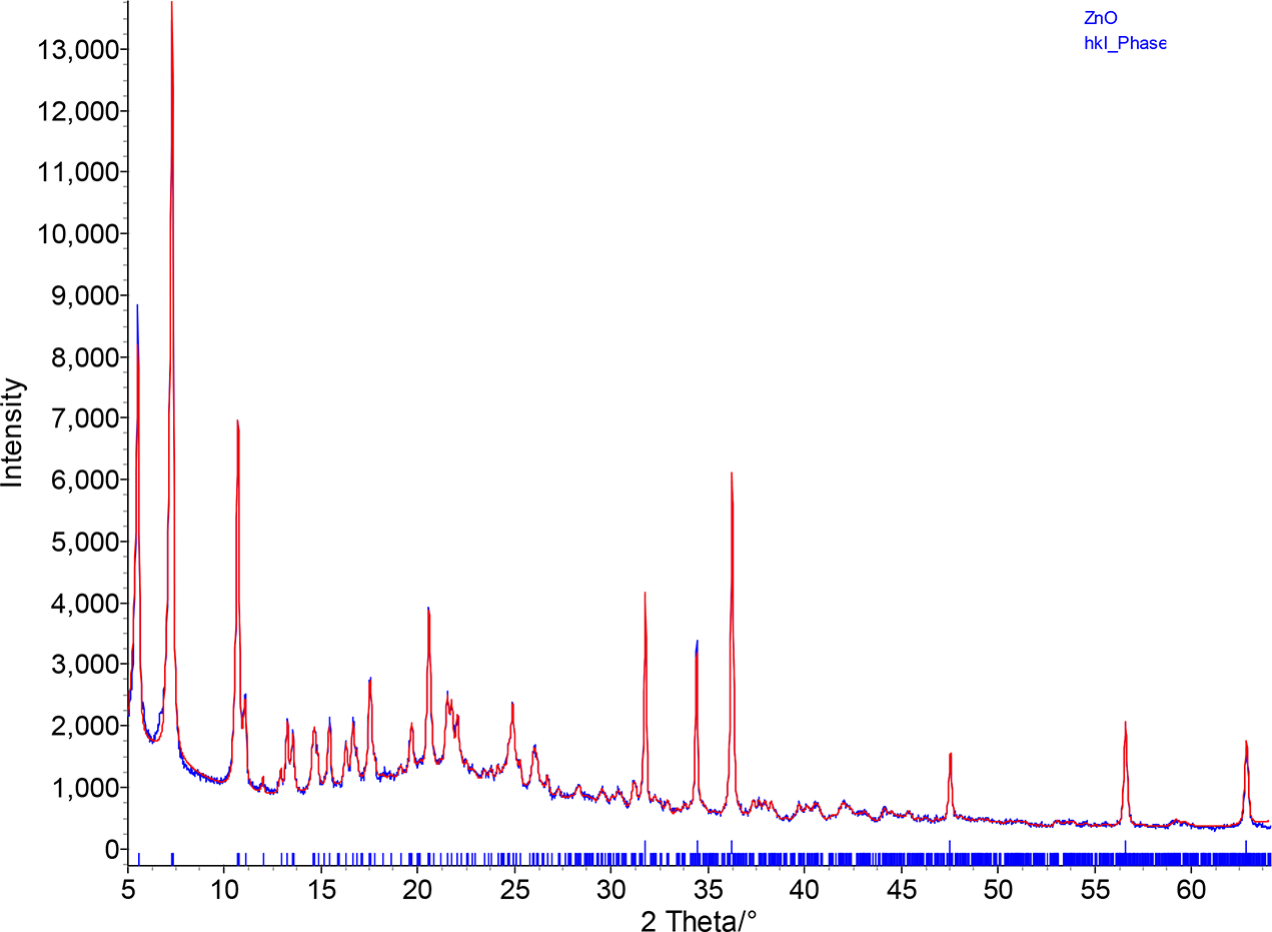
Simultaneous Rietveld (ZnO) and Pawley (intermediate **4**) refinement using powder X-ray diffraction data (λ = 1.5406 Å). Observed and calculated profiles are denoted by blue and red lines, respectively. Top reflection markers indicate ZnO and lower reflection markers intermediate **4**.

Intermediate **4**, which appears just before the formation of MOF-74, was found after 50 min of milling. The powder pattern of this intermediate has been successfully indexed (Table 1). Its unit-cell volume is 1.6 times larger than that of intermediate **3**. The presence of ZnO in the powder diffraction data indicates that this intermediate still has a lower Zn:H_4_DHTA ratio in comparison to MOF-74, but the composition of this final intermediate remains uncertain. Figure 5 shows a two-phase refinement using the powder diffraction data for this intermediate. A Rietveld refinement was used to model the ZnO phase, while a Pawley refinement was used for intermediate **4**. Searches for matching phases in the Cambridge Structural Database, as well as in the Northwestern Database of hypothetical MOFs,^43^ were unsuccessful. Attempts to solve the crystal structure from powder diffraction data or by growing single crystals were also unsuccessful.

### Mechanochemical Reaction Pathway

**Figure 6:**
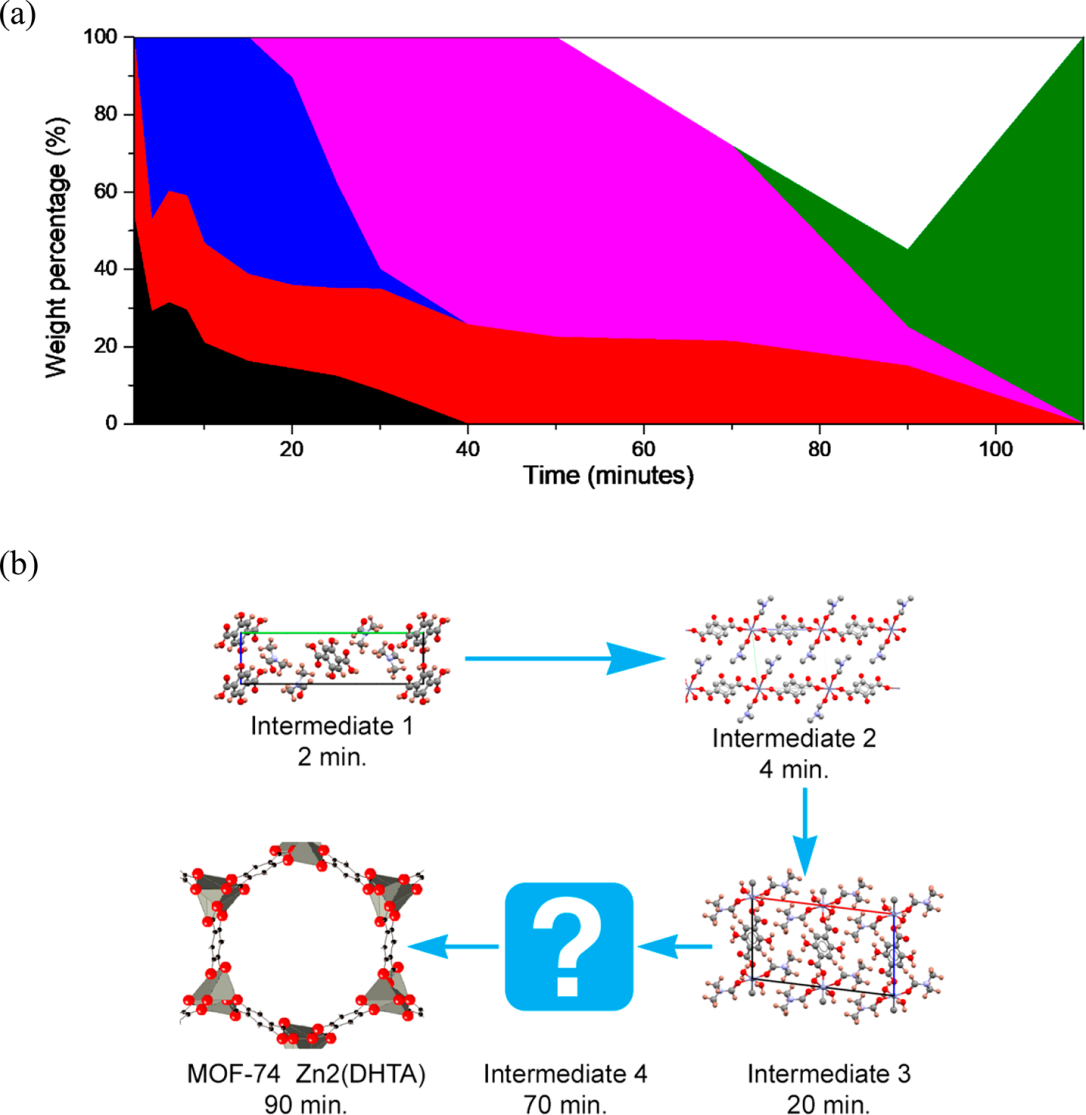
(a) Weight percentages for each phase as a function of milling time, for the mechanochemical reaction between H_4_DHTA and ZnO (1:2 ratio) in the presence of a small amount of DMF. The area under each color represents the weight percentage of each phase. Color key: intermediate **1**, black; ZnO, red; intermediate **2**, blue; intermediate **3**, pink; intermediate **4**, white; MOF-74, green. (b) Illustration of the phase evolution during the mechanochemical reaction between H_4_DHTA and ZnO in the presence of DMF.

Following the determination of the crystal structures of each intermediate, a series of multiphase Rietveld refinements were carried out using the diffraction data measured at different milling times. The weight percentage of each phase as a function of time was used to map out the changes as the reaction progressed (Figure 6a). The reaction pathway is illustrated graphically in Figure 6b. The final reaction product, MOF-74, has been characterized by powder X-ray diffraction, elemental analysis, thermogravimetric analysis, and FTIR (Supporting Information). The powder diffraction data for mechanochemically synthesized MOF-74 (Figure S4 in the Supporting Information) are in excellent agreement with those previously published for DMF-containing MOF-74 (CCDC code FIJDOS).^44^

A kinetic analysis is complicated by the presence of several intermediates, which make the application of conventional solid-state reactivity models^45^ nontrivial. An examination of the evolution of the weight fraction of ZnO (Figure 6a) suggests that the order of the reaction with respect to ZnO changes throughout the synthesis (e.g., transformation of intermediate **2** to intermediate **3** is likely to be of order zero for ZnO), but for a quantitative kinetic analysis it would be necessary to collect more data points in each intermediate region. It is interesting to note that the mechanochemical reaction described here entails the conversion of nonporous precursors (ZnO, *d* = 5.61 g cm^–3^; H_4_DHTA, *d* = 1.8 g cm^–3^) into a porous material, DMF-containing MOF-74, of density (*d* = 1.9 g cm^–3^)^44^ lower than that of ZnO. This occurs through the formation of nonporous intermediates, in contrast to the mechanochemical synthesis of MOFs such as ZIF-8, where porous phases are initially formed and prolonged milling leads to amorphization and the formation of dense phases.^22^ It has been previously reported that the mechanochemical synthesis of MOF-74 using water as a liquid additive^18^ also proceeds through the formation of a nonporous intermediate that converts into porous MOF-74.

**Figure 7:**
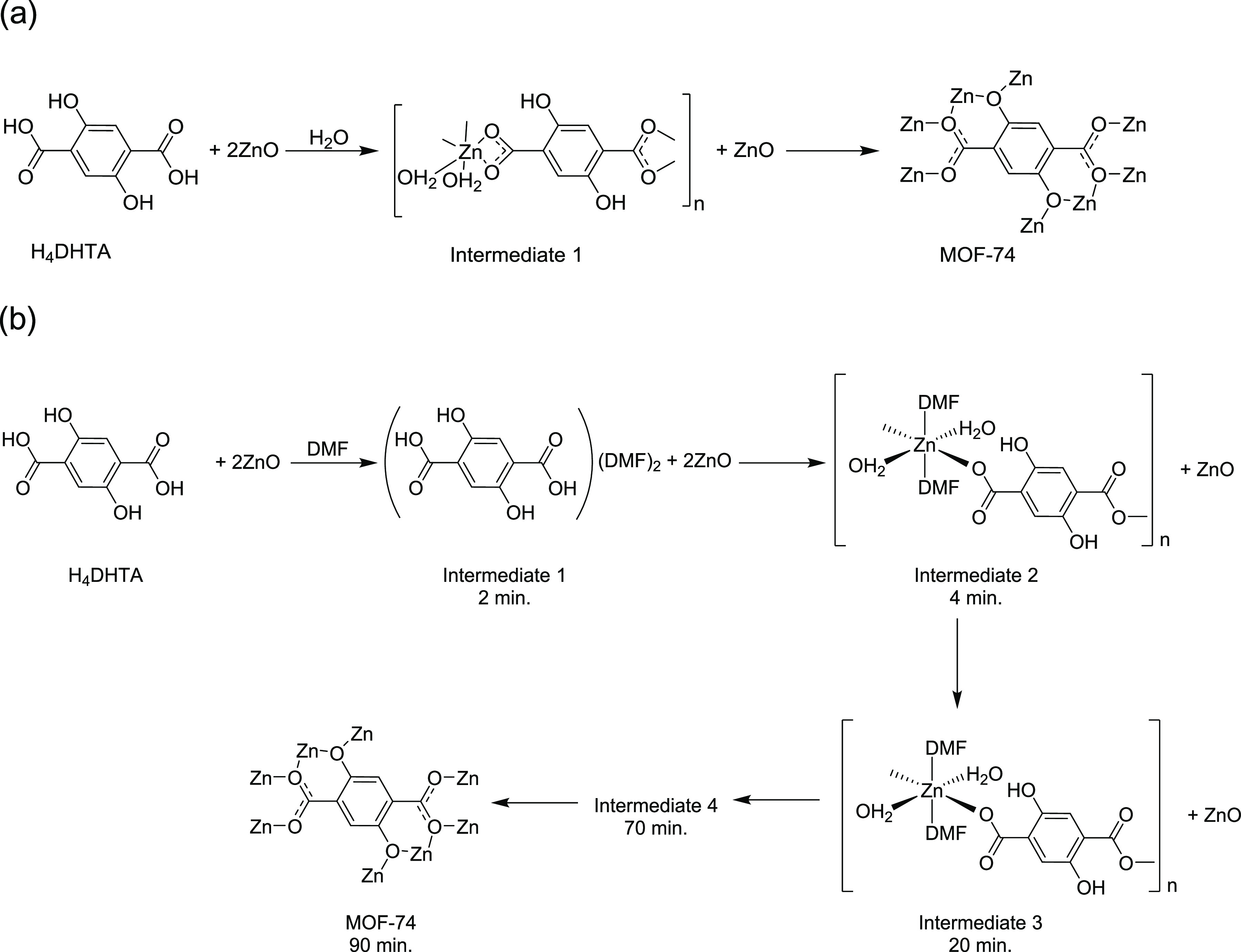
Reaction pathway for the mechanochemical reaction between H_4_DHTA and ZnO (1:2 ratio) in the presence of (a) a small amount of water^18^ and (b) a small amount of DMF.

This work demonstrates that the reaction pathway taken during a mechanochemical synthesis is dependent on which solvent is used to assist grinding (Figure 7). The mechanochemical synthesis of MOF-74 using water as a liquid additive had been previously investigated by Julien and co-workers,^18^ who found a single intermediate, Zn(H_2_DHTA)(H_2_O)_2_ (Intermediate **1** in Figure 7a).^46^ This intermediate, which can be described as consisting of one-dimensional chains of octahedrally coordinated Zn alternating with H_2_DHTA linkers, is markedly different from intermediates **2** and **3** found in this work. In the water intermediate (Figure 7a),^46^ the carboxylate groups coordinate in a bidentate manner (Zn–O distances of 2.00 and 2.57 Å) and the two H_2_DHTA linkers are arranged *cis* to each other. In contrast, in the DMF intermediates **2** and **3** (Figure 7b), the carboxylate denticity is only monodentate, and the linkers are arranged in a *trans* manner. The nature of the metal center also has an effect on the intermediates, as evidenced by recent work on the mechanochemical synthesis of mixed-metal MOF-74,^19^ where the coordination environment of water intermediates containing Mg^2+^, Co^2+^, or Ni^2+^ differs from that previously found for Zn^2+^.^18^

The final product, MOF-74, contains helical rods of composition [O_2_Zn_2_](CO_2_)_2_, with short Zn–Zn distances of ca. 3.1 Å, where each zinc metal center is octahedrally coordinated to one solvent molecule, three bridging carboxylates, and two hydroxyl groups from linker molecules.^44^ A significant structural rearrangement from the intermediates to the final product is required (Figure 7), as the Zn–Zn distances in both the water and DMF intermediates are approximately 8 Å. Moreover, ligand substitution is also needed, which in the case of the DMF intermediates **2** and **3** requires the removal of three solvent molecules (only one for the water intermediate). We might speculate that the DMF intermediate **4** involves the removal of some of the solvent molecules from the Zn coordination environment. MOF-74 appears after only 25 min of grinding when water is used, while with DMF 70 min is required, and another intermediate is formed prior to the conversion to the final product. For HKUST-1, it has been found that polar aprotic liquid additives such as DMF were less effective than protic liquids such as methanol,^25^ and a similar behavior may be at play here. Given that water and DMF have similar coordinating abilities to transition metals,^47,48^ the steric hindrance of the larger DMF molecule may also influence the nature of the intermediates and the reaction rate. The reaction pathway in the presence of both water and DMF involves intermediates in which the solvent is coordinated to the metal center, and therefore the use of noncoordinating or weakly coordinating solvents as liquid additives, which should lead to a different reaction pathway, might accelerate the formation of MOF-74.

## Conclusions

In summary, the investigation of the mechanism of mechanochemical reactions by powder X-ray diffraction enables the detection and isolation of new phases and can provide valuable information to establish the optimal reaction conditions. Our investigation of the mechanochemical synthesis of MOF-74 in the presence of DMF reveals a complex reaction pathway, involving four short-lived intermediate phases. Following structural charaterization of those intermediates, we can conclude that the synthesis of the highly porous MOF-74 proceeds via the formation of a nonporous solvate of H_4_HDTA and nonporous coordination polymers. The solvent used to assist grinding, DMF, is incorporated into the crystal structures of the intermediate phases, rather than simply filling the pores of the final MOF-74 product. We therefore conclude that the solvent plays a major role in the reaction pathway and the overall reaction rate. Further work on the effect of liquid additives, as well as on the effect of parameters such as the reaction temperature, will be of key importance to enable the rational design of mechanochemical syntheses of MOFs.

## Supporting Information Available

Stacked plot of I11 powder diffraction data, final rigid-body Rietveld refinements for intermediates **1** and **3**, a description of mechanochemical synthesis, and Rietveld refinement and characterization data for MOF-74 (PDF)

## Accession Codes

CCDC 2063893–2063895 contain the supplementary crystallographic data for this paper. These data can be obtained free of charge via www.ccdc.cam.ac.uk/data_request/cif, or by emailing data_request@ccdc.cam.ac.uk, or by contacting The Cambridge Crystallographic Data Centre, 12 Union Road, Cambridge CB2 1EZ, UK; fax: +44 1223 336033.
