# Supplementary material for: Insights into the Mechanochemical Synthesis of MOF-74
Source: Cryst Growth Des. 2021 Apr 27;21(5):3047–55. doi: 10.1021/acs.cgd.1c00213 (PMC8273859; doi:10.1021/acs.cgd.1c00213)
Supplement: Supplementary file 1 — cg1c00213_si_001.pdf [file cg1c00213_si_001.pdf]

Supporting Information for:

Insights into the Mechanochemical Synthesis of MOF-74

Jethro Beamish-Cook,[a] *Kenneth Shankland,*[a] *Claire A. Murray,*[b] *Paz Vaqueiro*[*a]

[a] School of Chemistry, Food and Pharmacy, University of Reading, Whiteknights, Reading RG6 6DX, England, United Kingdom

[b] Diamond Light Source, Harwell Science and Innovation Campus, Didcot OX11 0DE, United Kingdom

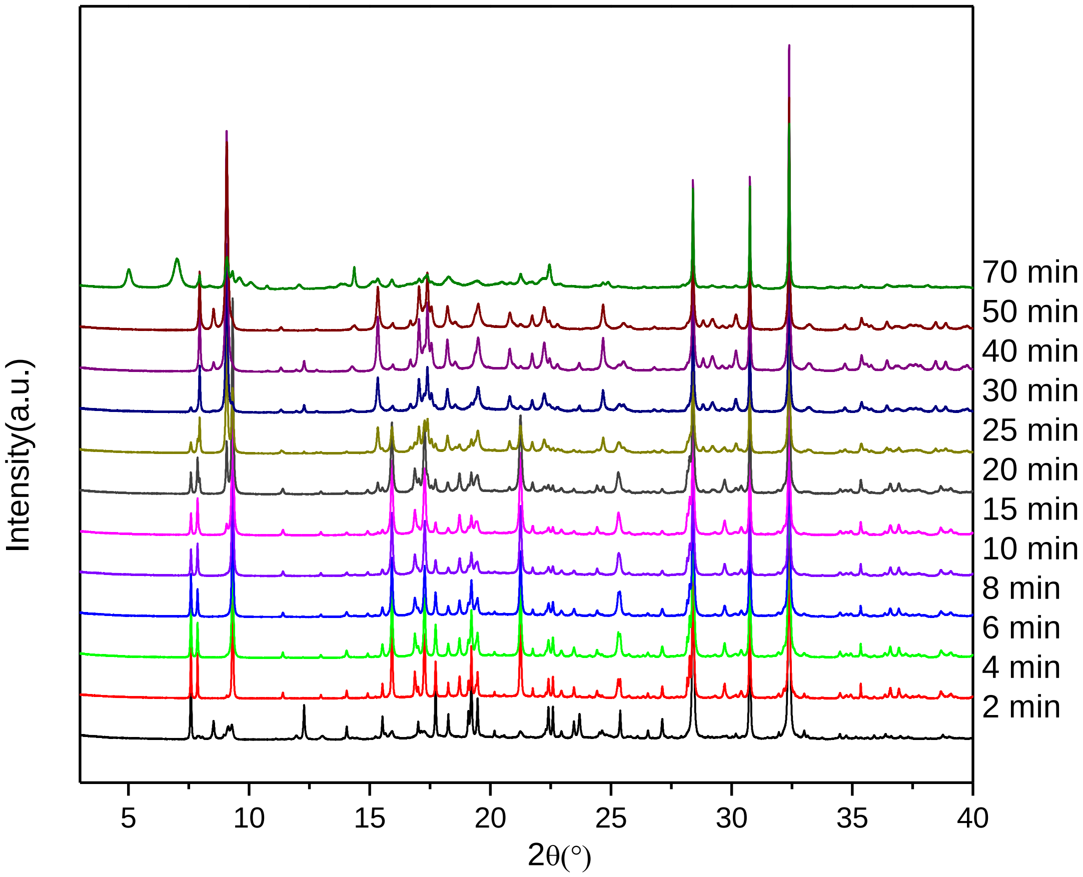

Figure S1. Powder diffraction data collected on I11 as a function of time.

Figure S2: Rietveld refinement using data collected on I11 for Intermediate 1 (blue tick marks), together with the contaminating phases ZnO (green tick marks) and intermediate 2 (red tick marks) over the ranges (*a*) 5-80° (*b*) 5-40° and (*c*) 40-80° 2θ

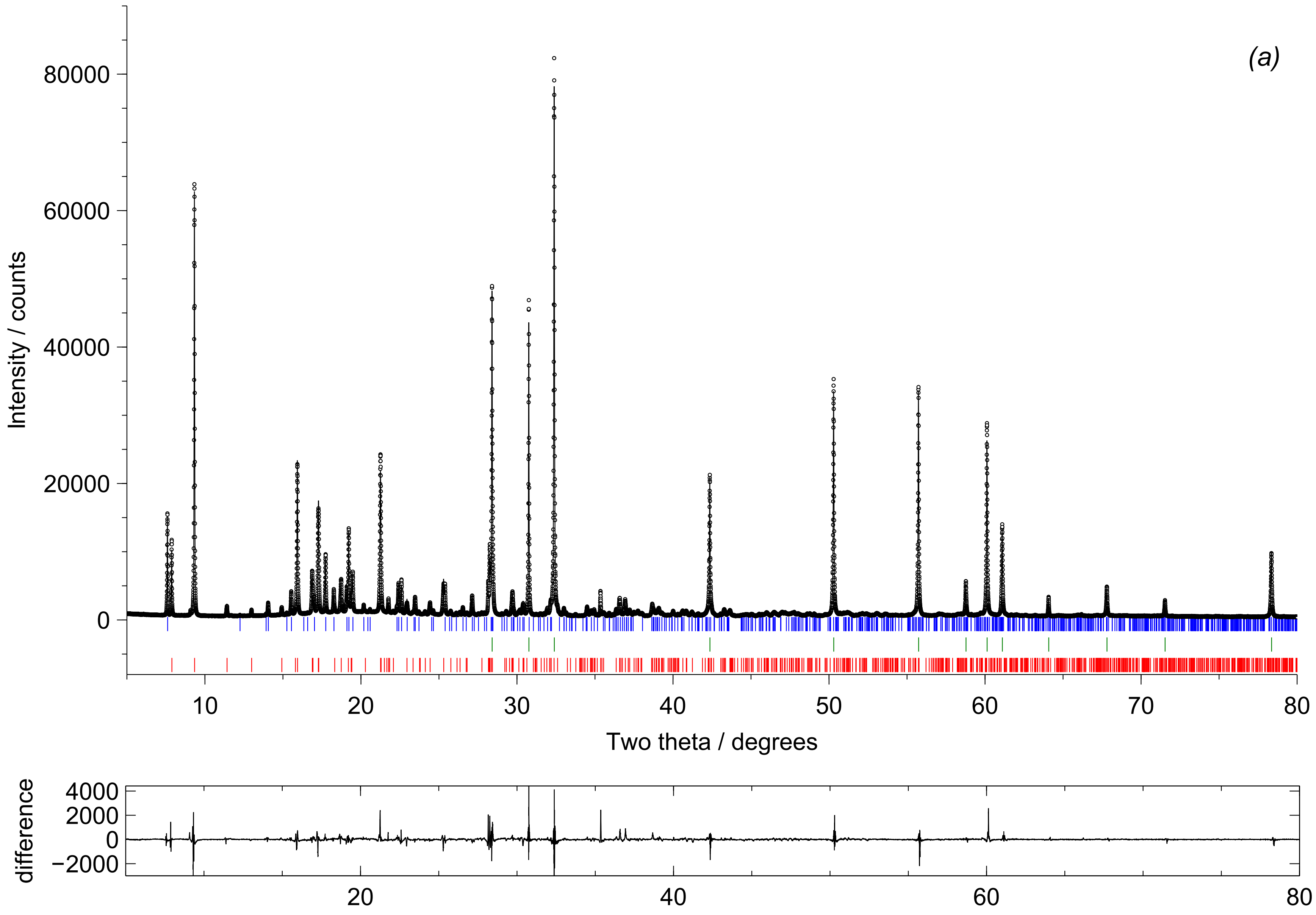

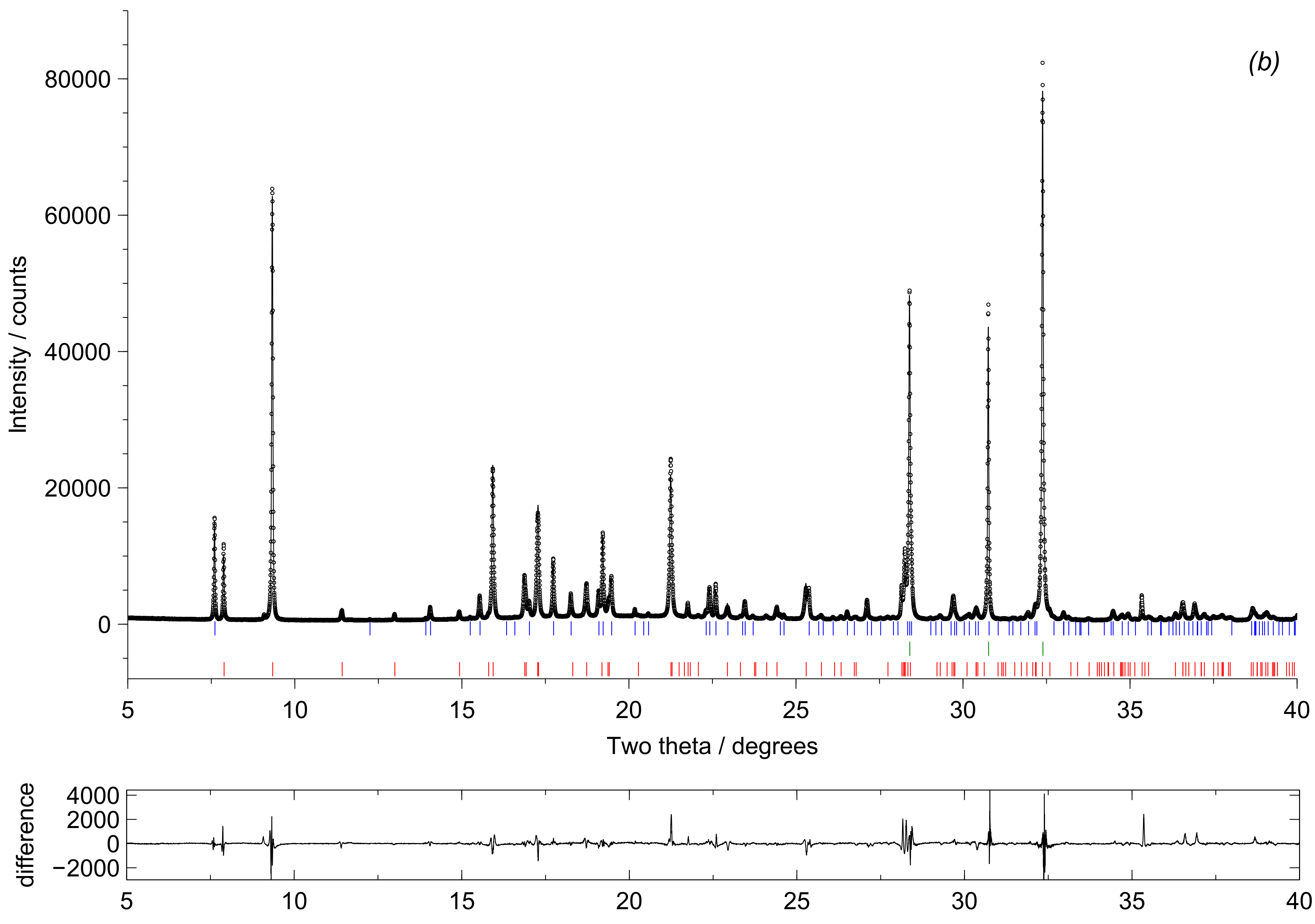
(b)
Intensity / counts
0
20000
40000
60000
80000
5
10
15
20
25
30
35
40
Two theta / degrees
difference
4000
2000
0
−2000
5
10
15
20
25
30
35
40

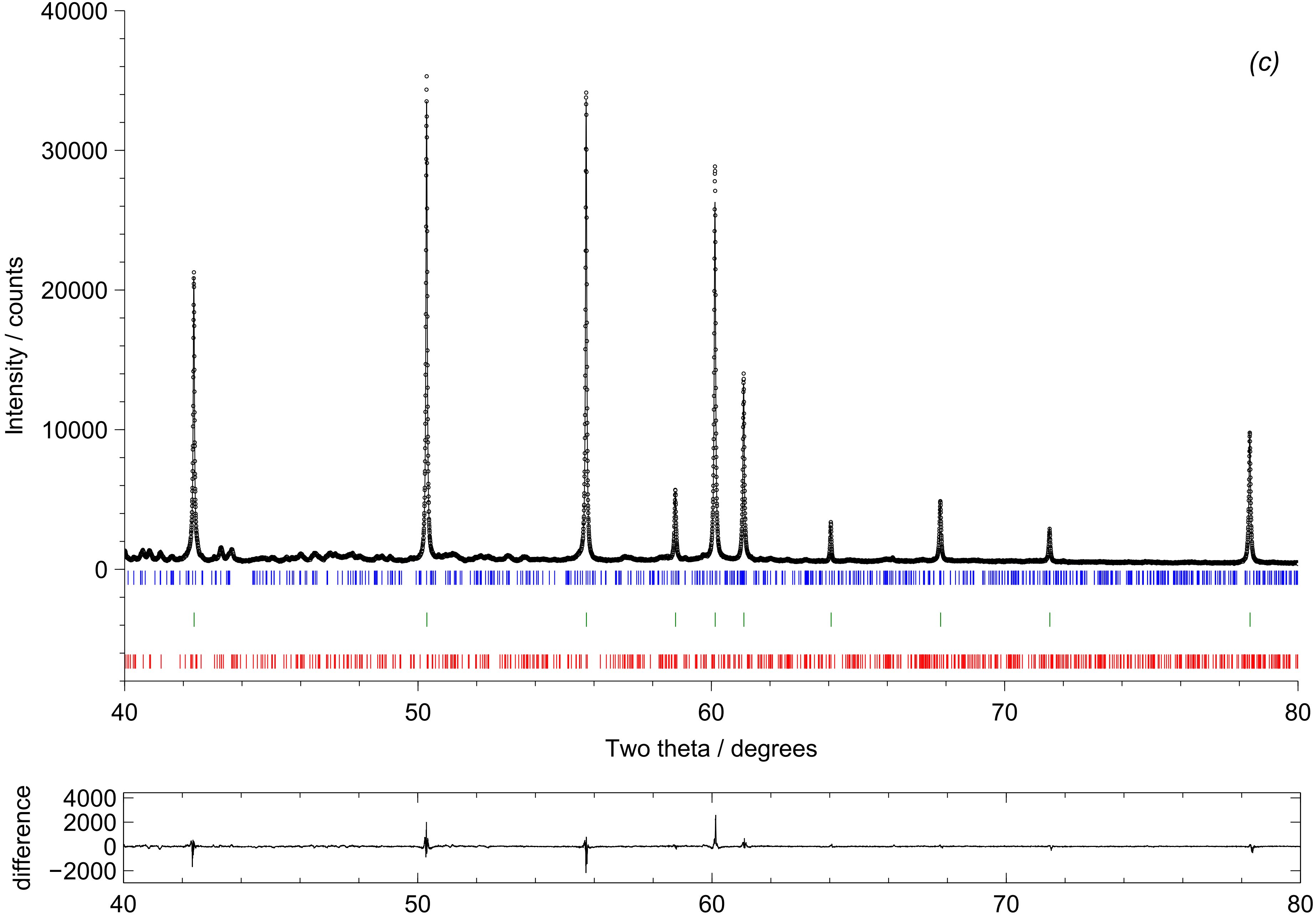
(c)
Intensity / counts
40000
30000
20000
10000
0
40
50
60
70
80
Two theta / degrees
difference
4000
2000
0
−2000
40
50
60
70
80

Figure S3: Rietveld refinement using data collected using data collected on I11 for Intermediate 3 (blue tick marks) and contaminating phase Zn(H_2DHTA)(H_2O)$_2$,CCDC refcode ODIPOH (red tick marks) over the ranges (*a*) 5-60° (*b*) 5-30° and (*c*) 30-60° 2θ.

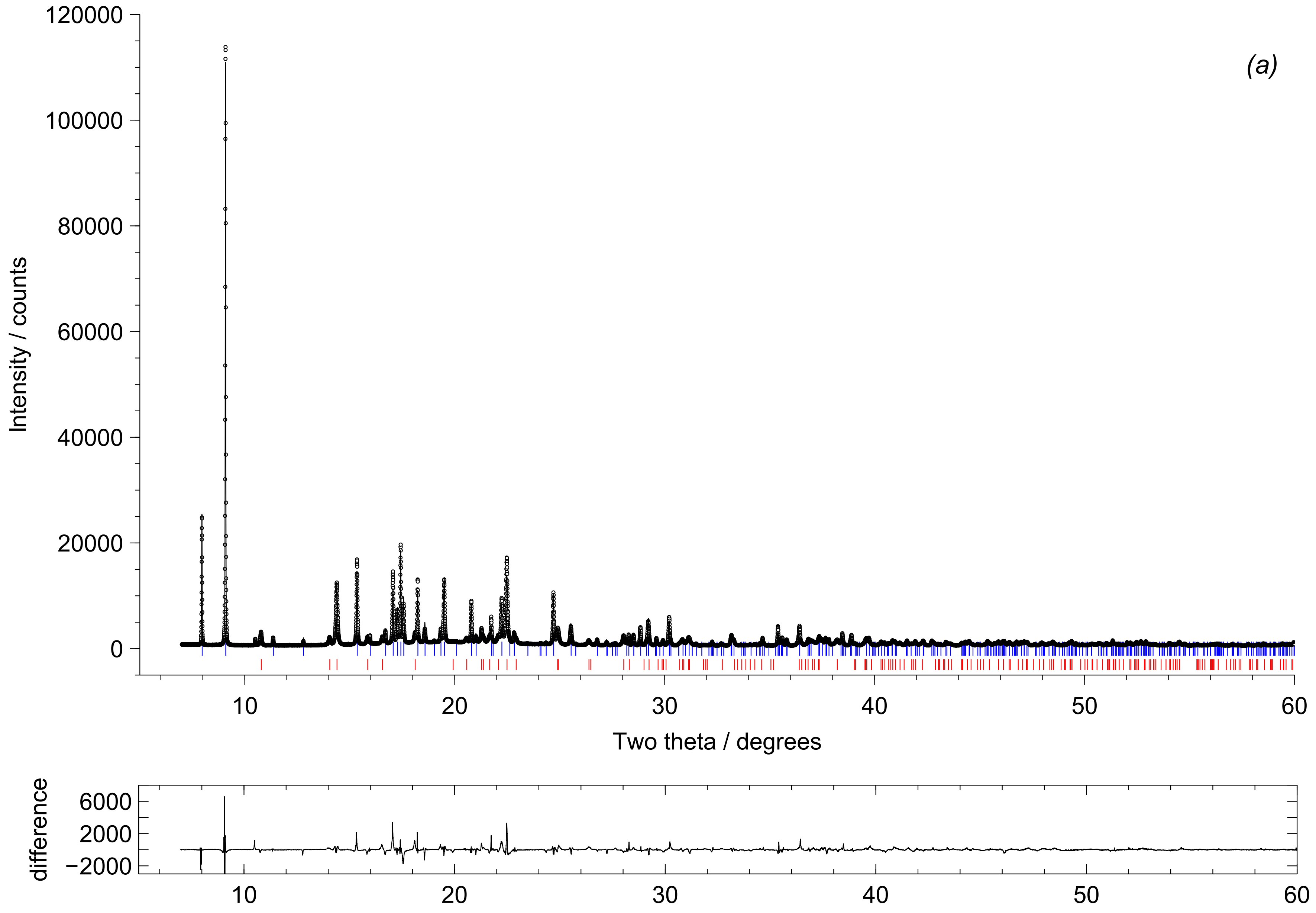

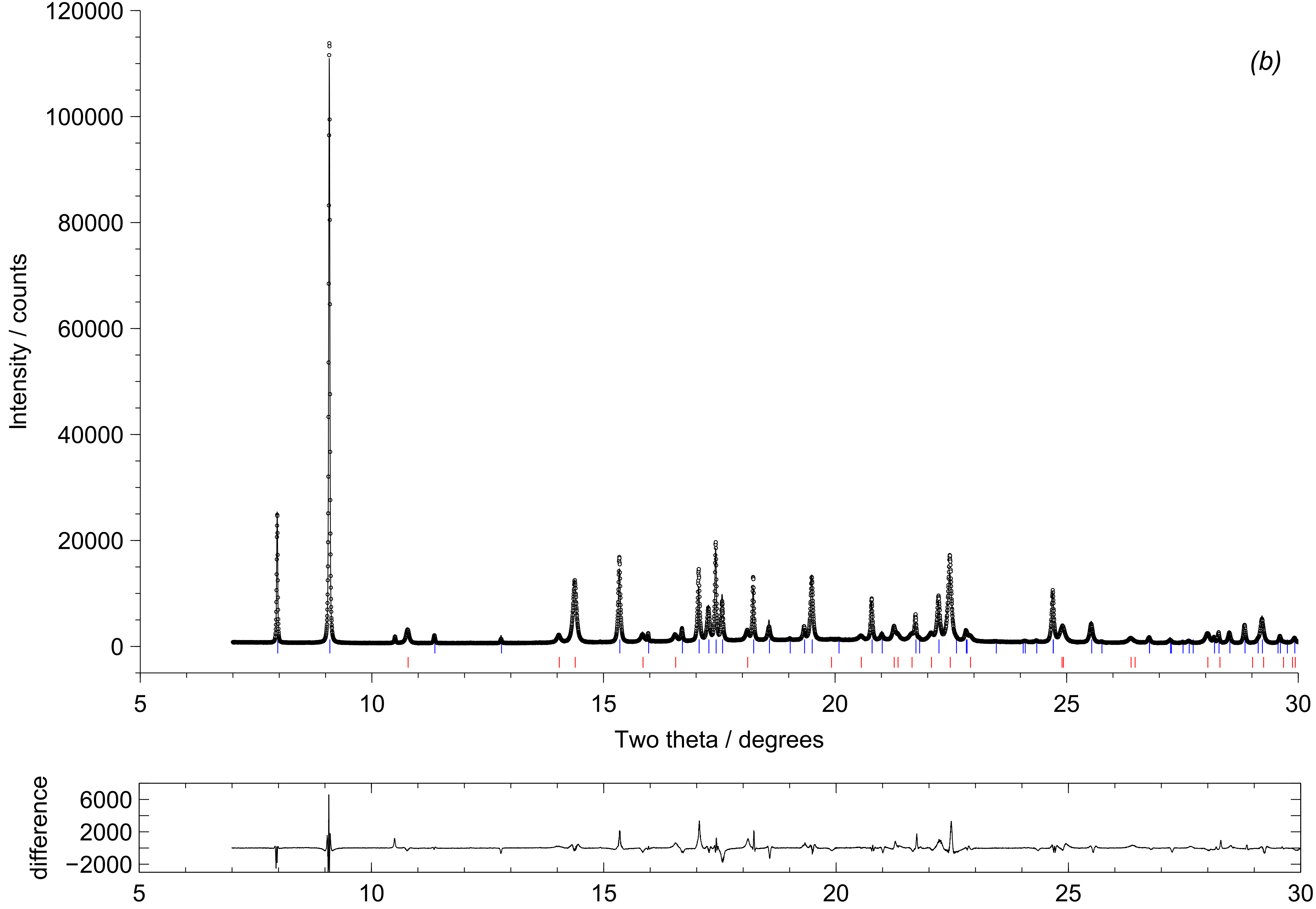
(b)
Intensity / counts
Two theta / degrees
difference
120000
100000
80000
60000
40000
20000
0
5
10
15
20
25
30
6000
2000
−2000

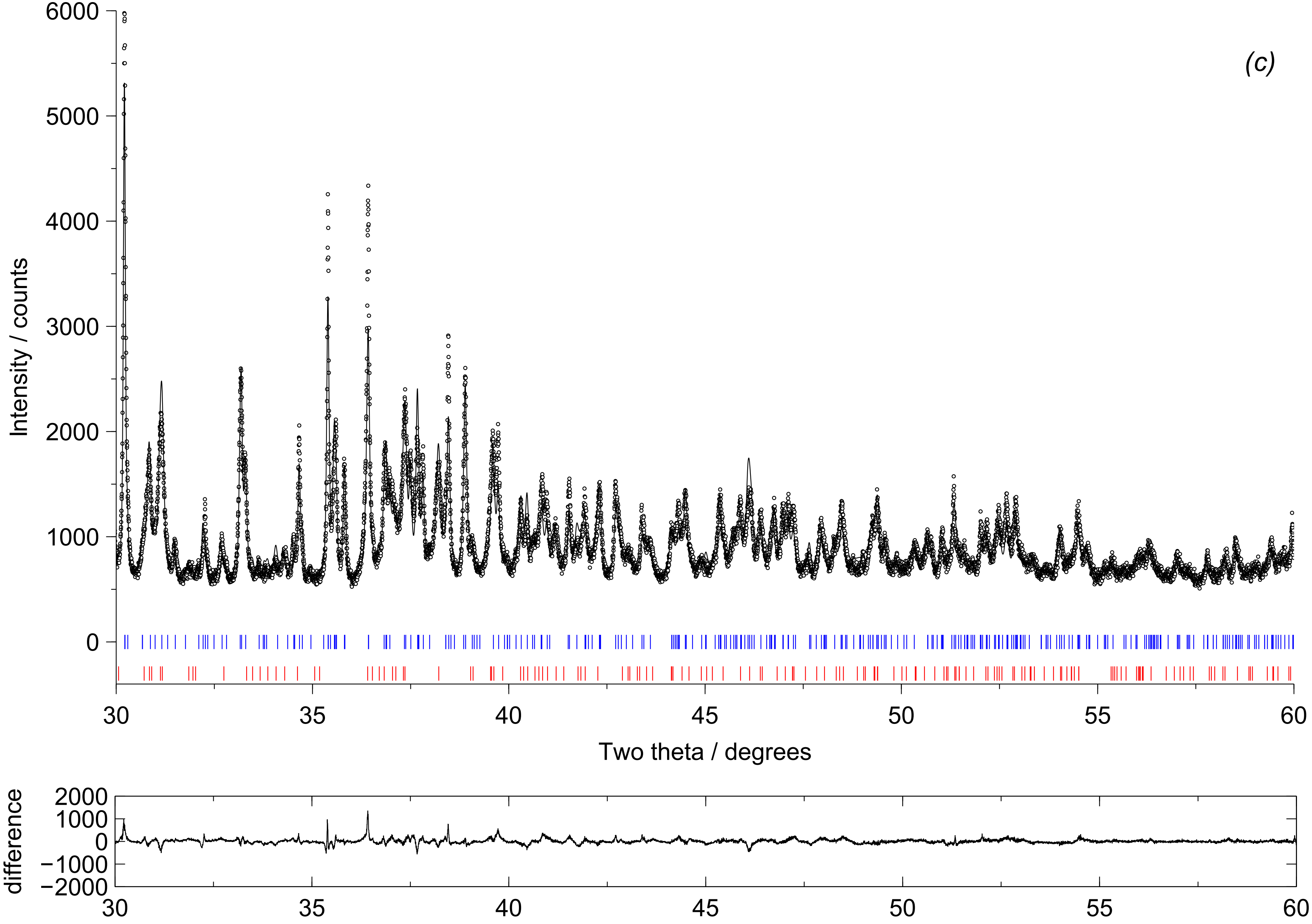
(c)
Intensity / counts
6000
5000
4000
3000
2000
1000
0
30
35
40
45
50
55
60
Two theta / degrees
difference
2000
1000
0
−1000
−2000

Uninterrupted mechanochemical synthesis of MOF-74

179 mg of ZnO (2.2 mmol), 220 mg of DHTA (1.1 mmol) and 300 μL of DMF were added to a 25 mL stainless steel milling jar, together with a single 7 g stainless steel ball. The jar was sealed, and the materials milled for 90 minutes at a rate of 30 Hz using a Retsch MM400 shaker-type mixer mill. The product was washed with 5 mL DMF to remove trace amounts of the starting materials. The estimated yield of the reaction was greater than 90%.

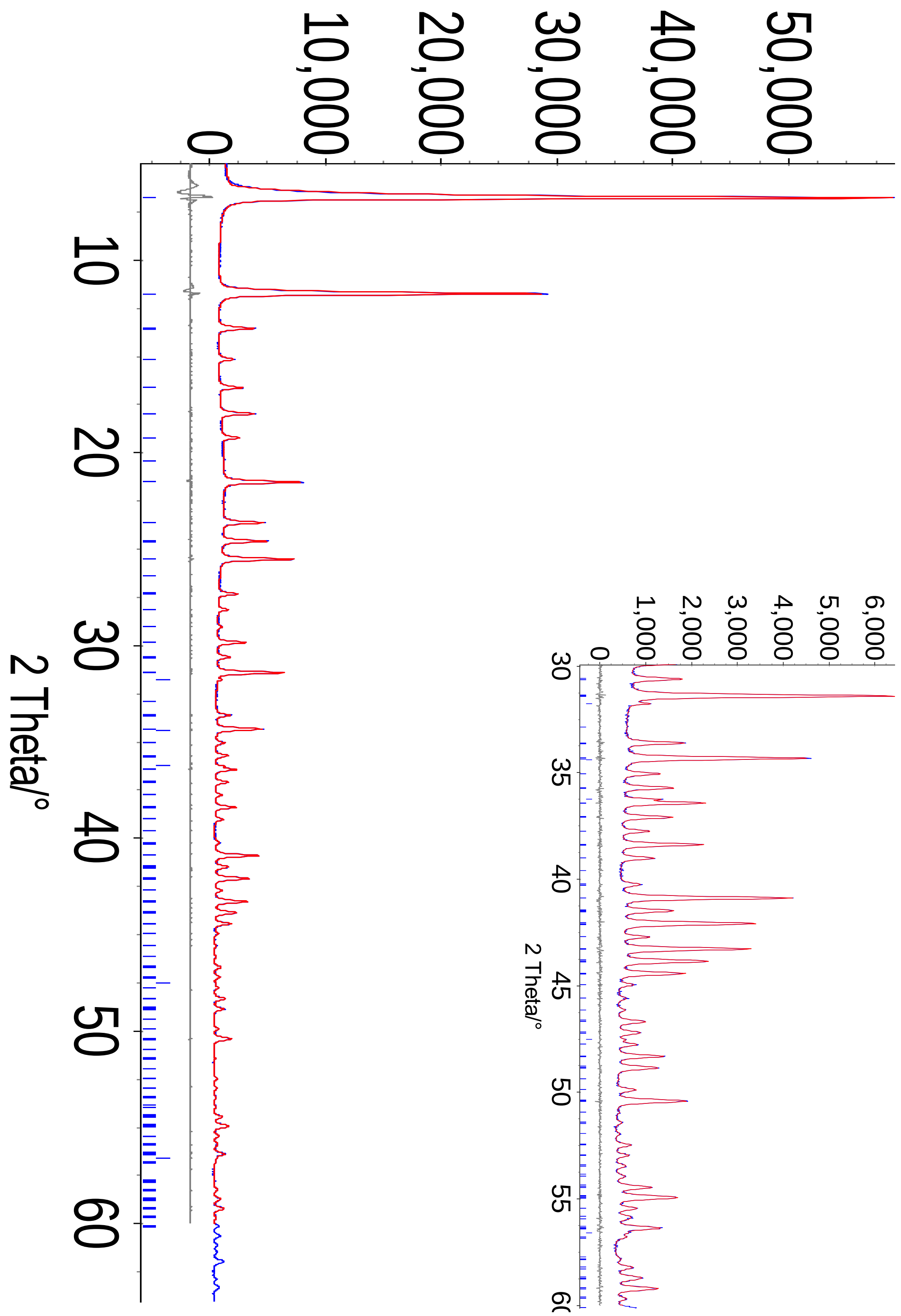

Figure S4. Rietveld refinement using data collected using a Bruker D8 diffractometer (λ = 1.5406 Å) for mechanochemically-synthesised MOF-74. Observed, calculated and difference profiles are denoted by blue, red and grey lines, respectively. Lower markers correspond to MOF-74 reflections and upper markers to ZnO. Atom coordinates, which were not refined, were obtained from the cif CCDC refcode FIJDOS.

Table S1. Elemental analysis of MOF-74, with a theoretical formula of $Zn_2(DHTA)(DMF)_{0.5}(H_2O)_4$

Element	Calculated (%)	Experimental (%)
C/%	26.32	26.95
H/%	3.12	3.11
N/%	1.62	1.73

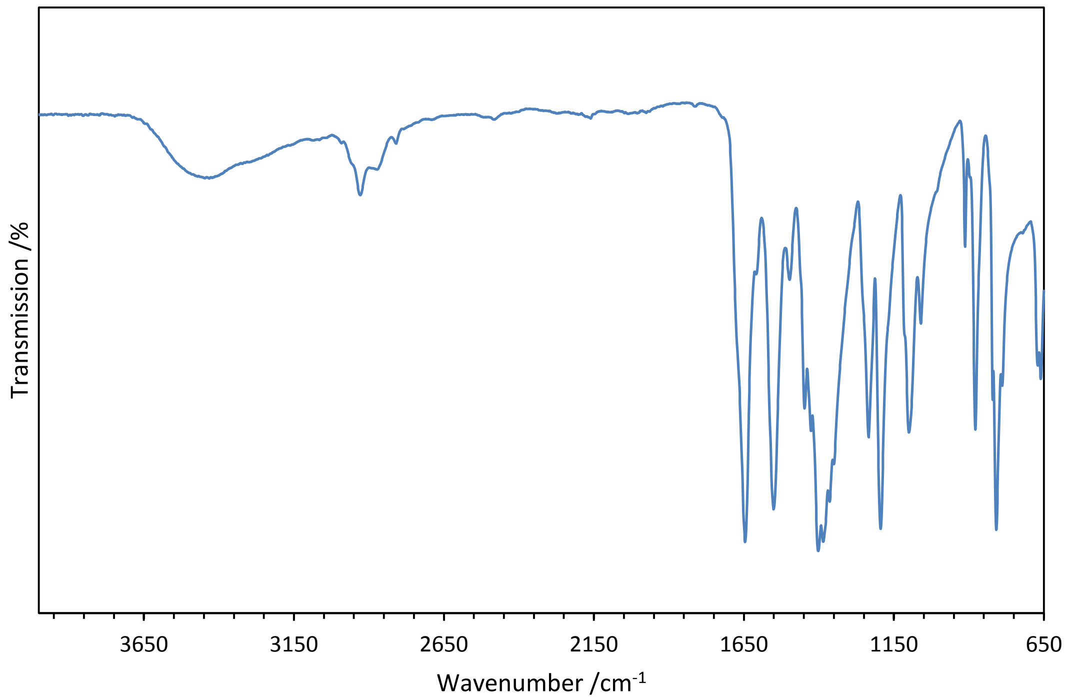

Figure S5. FTIR spectrum of MOF-74. Peaks (cm^{-1}): 809 (s), 879 (m), 912 (w), 1044 (m), 1060 (w), 1100 (m), 1194 (s), 1234 (m), 1400 (s),1497 (w), 1551 (s), 1646 (m), 2868 (w), 2930 (w), 34 (br).

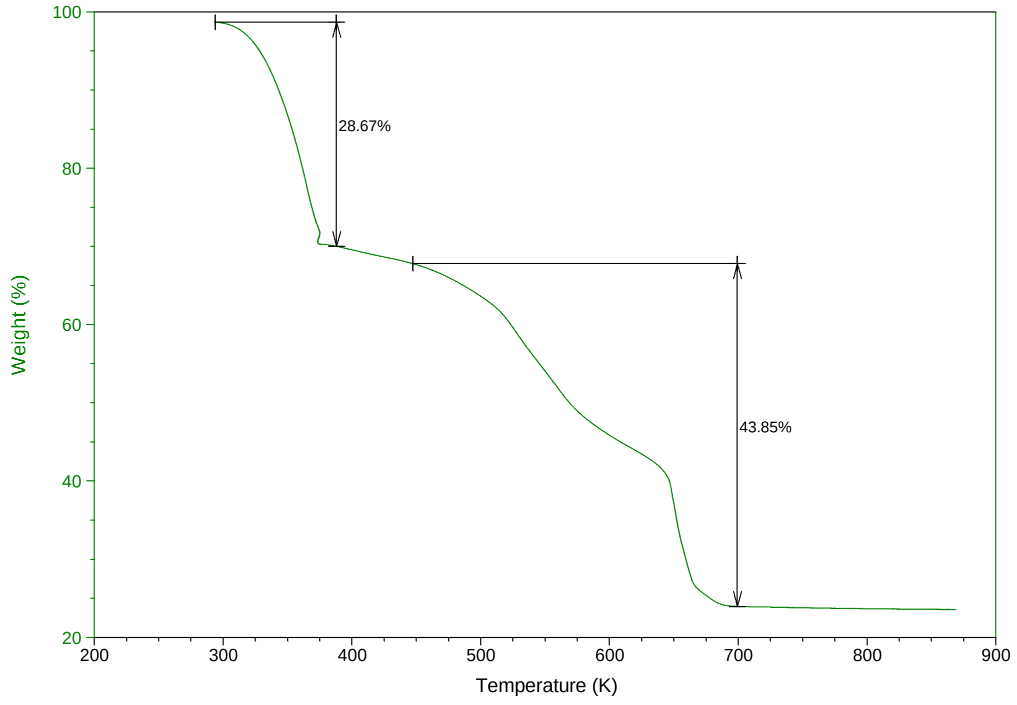

Figure S6. Thermogravimetric analysis data for MOF-74 collected under a flowing N_2 atmosphere.
